# Perceptual Evaluation of 3D-Printed Typodont Teeth with Comparable Cutting Forces

**DOI:** 10.3390/jfb17090471

**Published:** 2026-09-17

**Authors:** Alexander Jon Cresswell-Boyes, Aylin Baysan, Graham Roy Davis

**Affiliations:** 1 Peninsula Dental School, University of Plymouth, Plymouth PL4 8AA, UK; 2 Dental Physical Sciences Unit, Centre for Oral Bioengineering, Institute of Dentistry, Barts and the London School of Medicine and Dentistry, Queen Mary University of London, London E1 4NS, UK; g.r.davis@qmul.ac.uk; 3 Centre for Oral Bioengineering, Institute of Dentistry, Barts and the London School of Medicine and Dentistry, Queen Mary University of London, London E1 2AD, UK; a.baysan@qmul.ac.uk

**Keywords:** dental education, 3D printing, simulation-based education, typodont, haptics, surface roughness

## Abstract

**Background**: Pre-clinical training in endodontics relies heavily on typodont teeth to develop operative skills before patient care, but conventional polymer-based typodonts differ from human teeth in their cutting response, requiring greater force and producing an unrealistic cutting experience that often dissatisfies students. **Methods**: This study evaluated four alternative 3D-printed typodont materials—15 wt.% carbonated hydroxyapatite, 15 wt.% Puraflake^®^ (glass flake filler), 15 wt.% zinc oxide, and a urethane/triethylene glycol dimethacrylate resin composite—based on their cutting force, post-cut surface roughness, and perceived cutting feel relative to extracted enamel, assessed via a five-point Likert scale questionnaire completed by 46 (*n* = 46) fourth- and fifth-year dental students. **Results**: No statistically significant differences in cutting force were detected between the four printed materials and extracted enamel, a finding confirmed using formal two one-sided equivalence testing (TOST); larger confirmatory studies would allow this equivalence to be established with greater statistical precision. Perception scores differed significantly between materials, and fifth-year students rated the dental resin composite significantly more favourably than fourth-year students, with only the composite effect surviving stringent Bonferroni correction across all four material comparisons, underscoring the reliability of this specific finding. Post-cut surface roughness showed a strong, statistically robust monotonic association with perception scores at the individual-respondent level (Page’s L trend test, *n* = 46, *p* < 0.001), suggesting that surface behaviour during material removal may contribute to perceived haptic similarity. **Conclusions**: Matching cutting force alone does not guarantee perceptual equivalence. Among the materials tested, the dental resin composite most closely approximated extracted-enamel surface roughness, received the most favourable and consistent perception scores, and is recommended as the preferred material for pre-clinical typodont fabrication where accessible. These 3D-printed typodonts offer a standardised, openly documented alternative to commercial typodonts for pre-clinical training, with digital models and manufacturing workflows openly available via an institutional platform (TactiTooth).

## 1. Introduction

Pre-clinical simulation is central to undergraduate dental education, with typodont teeth widely used to develop psychomotor skills prior to patient care. Extracted human teeth remain the reference standard due to their anatomical complexity and authentic cutting response, but their use is increasingly constrained by availability, ethical approval and standardisation challenges [1,2,3,4,5,6,7]. Conventional polymer-based typodonts address these logistical limitations but differ substantially from natural dental tissues in their mechanical behaviour, requiring greater cutting forces than extracted enamel and producing a cutting experience that students perceive as unrealistic [1,2,8,9].

In response to these limitations, a range of modified and customised typodont systems have been proposed, including artificial caries models, patient-specific typodonts derived from clinical imaging, and 3D-printed replicas fabricated using cone-beam computed tomography or X-ray microtomography with high geometric fidelity [10,11,12,13,14,15,16,17,18,19]. These approaches frequently report improved student engagement and perceived realism [11,12,13], but many studies conflate visual realism, anatomical fidelity, and haptic feedback, making it difficult to isolate the specific contribution of material behaviour to perceived realism [12,14].

Despite these advances, the majority of 3D-printed typodont teeth continue to be fabricated from commercially available photopolymer resins whose mechanical properties differ markedly from those of enamel and dentine [15,19]. While anatomical accuracy has been rigorously validated, discrepancies in material behaviour are frequently acknowledged as a limitation [20].

Recent work has sought to quantify the cutting behaviour of dental tissues and typodont materials using objective mechanical measurements. Cutting force has been shown to be a meaningful indicator of material response during drilling and instrumentation [21,22,23]. Enamel cutting behaviour is known to be complex, reflecting its anisotropic microstructure, brittle–plastic fracture mechanisms and sensitivity to cutting direction and tool geometry [23,24,25]. Importantly, cutting behaviour is not governed by hardness alone, and materials with similar bulk properties may exhibit markedly different responses during machining [21,26]. These findings underscore the challenge of reproducing dental tissue behaviour using simplified material systems.

In parallel, studies in machining science and haptics have demonstrated that human operators are sensitive not only to mean cutting force but also to fluctuations in force, vibration and acoustic feedback during material removal [27,28,29]. Perceived resistance is therefore a multisensory construct, shaped by dynamic interactions between the tool, material and operator. In the context of dental education, novice learners may be particularly reliant on immediate surface-level cues and consistency of resistance, whereas experienced clinicians integrate tactile feedback over longer time scales and depths [30]. This distinction suggests that material equivalence defined solely by mechanical metrics may not guarantee perceptual equivalence for undergraduate students.

Previous work by the present authors has demonstrated that 3D-printed typodont teeth can be engineered to exhibit cutting forces comparable to extracted enamel through the use of resin composite formulations incorporating particulate fillers [31,32,33]. These studies established that relative mechanical properties, rather than absolute hardness, govern cutting response, and reported that force-matched cutting behaviour does not guarantee perceptual equivalence [33]. The present study extends this earlier work in several respects. Building methodologically on this prior work, it characterises four alternative printable materials not previously evaluated by the group and stratifies student perception by year group. Its principal novel contribution is establishing, for the first time and at the level of individual student responses rather than material averages, a statistically robust relationship between post-cut surface roughness and perceived haptic similarity, providing a specific, testable candidate explanation for the perceptual differences previously only observed descriptively [33]. The resulting digital models and manufacturing workflows are also disseminated via the TactiTooth platform to support reproducibility.

The aim of this study was therefore to evaluate alternative composite materials for the fabrication of 3D-printed typodont teeth that exhibit cutting forces comparable to extracted enamel, and to assess undergraduate dental students’ perceptions of their haptic similarity. In addition, this work seeks to support reproducibility and educational translation through open-access dissemination of the digital models and manufacturing workflows developed.

## 2. Materials and Methods

### 2.1. Ethical Approval and Data Protection

Ethical approval for the study was obtained from the Queen Mary Ethics of Research Committee (QMER20.586/2021, 8 December 2021), with the feedback being collected anonymously using the Online Surveys (formerly BOS; JISC, Bristol, UK) platform. Students consented before participation and were informed that their participation was not linked to their academic progress. Data was processed and stored following current data protection laws.

Extracted human teeth used for cutting-force and surface-roughness measurements were obtained from a human tissue bank under ethical approval from the Queen Mary Research Ethics Committee (QMREC2014/17, 20 March 2014), as previously described [31]. Non-carious mandibular molars were selected, with enamel and dentine surfaces prepared for cutting under the protocol described below. A commercial Frasaco typodont (ANA-4, Frasaco GmbH, Tettnang, Germany) was used as the comparator typodont material.

### 2.2. The Geometry of the 3D-Printed Typodont Teeth

**Figure 1:**
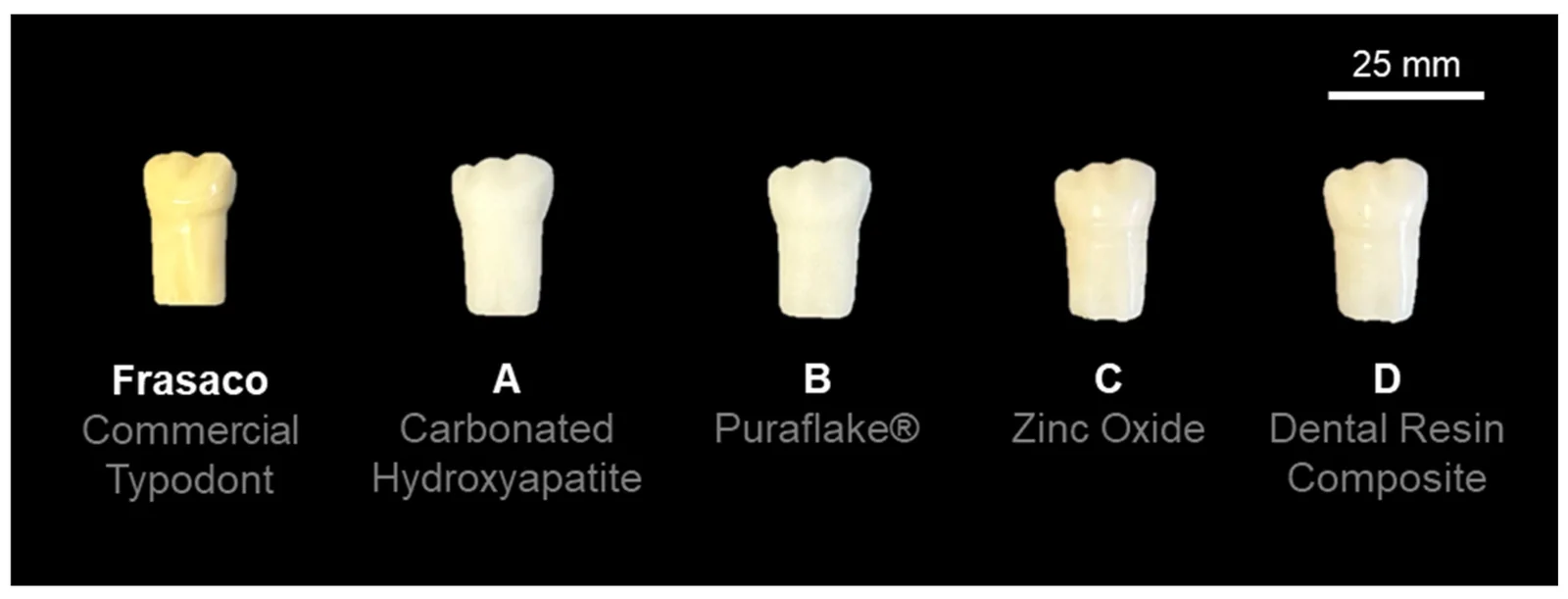
Typodont tooth specimens evaluated in this study. From left to right: commercial Frasaco typodont; 3D-printed typodont fabricated with 15 wt.% carbonated hydroxyapatite composite (Tooth A); 15 wt.% Puraflake^®^ composite (Tooth B); 15 wt.% zinc oxide composite (Tooth C); dental resin composite (Tooth D). All 3D-printed teeth were fabricated from the Frasaco mandibular first molar geometry acquired by X-ray microtomography [14].

The geometry of a mandibular first molar typodont tooth (Frasaco GmbH, Tettnang, Germany) was acquired using the MuCAT2 X-ray microtomography scanner [14,34]. The specimen was scanned at 40 kV and 405 μA with a voxel size of 15 μm. The reconstructed datasets were segmented and converted into a surface mesh before being exported as an *.stl file format for subsequent 3D printing. This approach ensured that both external morphology and internal structure were representative of commonly used educational typodont teeth. The resulting printed specimens are shown alongside the commercial Frasaco comparator in Figure 1.

### 2.3. Production of the 3D-Printed Typodont Teeth

Four alternative materials were evaluated in this study, selected both to explore a range of filler types previously shown to influence cutting response and because their cutting forces were expected to approximate extracted enamel based on our prior work [31,32,33]. The materials investigated were:

- A 15 wt.% carbonated hydroxyapatite, 85 wt.% photopolymer resin (Anycubic 405 nm Rapid Resin, Anycubic, Shenzhen, China). The carbonated hydroxyapatite was produced as outlined by Landi et al. [35].
- A 15 wt.% Puraflake^®^ (Glassflake Ltd., Leeds, UK) and 85 wt.% photopolymer resin.
- A 15 wt.% zinc oxide (Merck, Darmstadt, Germany) and 85 wt.% photopolymer resin.
- Dental composite resin (adapted from Sa et al. [36]); 60 wt.% urethane dimethacrylate (UDMA), 35 wt.% triethylene glycol dimethacrylate (TEGDMA), 1 wt.% Lucirin, and 4 wt.% zinc oxide. All reagents were sourced from Merck (Darmstadt, Germany).

All particulate fillers were mechanically mixed with the base photopolymer resin until a homogeneous suspension was achieved; homogeneity was verified by repeat sampling. Suspensions were stored in opaque containers to prevent premature curing. Typodont teeth were fabricated using a stereolithography-based 3D printer (Anycubic Mono 4K, Anycubic, Shenzhen, China), with a layer thickness of 50 μm and an exposure time of 8 s per layer. Printed models were washed in 90% ethanol for 20 min (Formlabs Wash; Formlabs, Somerville, MA, USA) to remove uncured resin, then post-cured using a Formlabs Cure (Formlabs, Massachusetts, USA) unit for 30 min at 60 °C.

As these formulations were produced in-house rather than sourced commercially, no manufacturer datasheet values were available. Degree of conversion, Vickers microhardness, flexural modulus, and filler particle size/morphology were characterised for each formulation; the full methodology, instrumentation, and results are provided in the Supplementary Material and Supplementary Table S1.

### 2.4. Haptic Response Measurements

Utilising the method established in Cresswell-Boyes, Davis, Krishnamoorthy, Mills and Barber [31], the 3D-printed typodont teeth, extracted enamel and dentine specimens, and commercial (Frasaco) typodont teeth were embedded in acrylic blocks (Kemdent Simplex Rapid, Associated Dental Products Ltd., Swindon, UK) and mounted to a 3-axis load cell (Model 3A60A, Interface Force Measurements Ltd., Crowthorne, UK). A high-speed dental handpiece (TE-95 BC Alegra Dental Air Rotor Handpiece, The W&H Group, Bürmoos, Austria), fitted with a cylindrical diamond bur (111-012 M, Dentsply Sirona, Charlotte, NC, USA), was run at a constant rotational speed of 40,000 rpm and driven into each specimen at a linear feed rate of 1 mm/s to a cut depth of 1 mm, with each cut taking approximately less than 45 s. A new bur was used for each specimen. Water irrigation was used throughout cutting. Load data were recorded in the mesiodistal (X) direction at a sampling rate of 250 Hz, logged using a signal amplifier (ME-Meβsysteme GmbH, Hennigsdorf, Germany) and software (GSVmulti Version 1.40, 2018; ME-Meβsysteme GmbH, Hennigsdorf, Germany). The mean cutting force for each specimen was calculated as the 5th–95th percentile trimmed average of the recorded load trace, consistent with our prior work [31].

### 2.5. Surface Characterisation

Post-cut surface roughness was quantified using a white light profilometer (ProScan 2000, Scantron Industrial Products Ltd., Taunton, UK), a non-contact optical device that utilises white light to measure surface distance. Each post-cut surface was scanned over a 1 mm × 1 mm area with a 10 μm sampling interval in X and Y, and a Gaussian cut-off filter of λc = 0.8 mm was applied. Form was removed from the curved cut surface using auto-level/best-fit plane subtraction, followed by the ProScan roughness filter. Measurements were collected and analysed using ProScan 2000 software (Version 2.1.1.8+, 2003; Scantron Industrial Products Ltd., Taunton, UK). Eight repeat measurements were obtained per material group, with each measurement taken on a separate specimen to ensure independence.

### 2.6. Questionnaire Design and Distribution

A questionnaire was administered to fourth- and fifth-year dental students who were participating in the Clinical Skills Laboratory-based Endodontics Speciality course. Students performed standardised Class I cavity preparation on each of the four typodont materials, presented in a randomised order; no extracted-tooth reference specimen was provided during the session, so ratings relied on students’ recalled experience of cutting enamel. The Likert scale questionnaire consisted of four perception-based questions and two closed-ended questions (Supplementary Figure S1). The questionnaire aimed to compare the 3D-printed typodonts with extracted enamel. Numerical values were assigned to the Likert scale questions for data analysis, with “strongly agree” (1), “agree” (2), “neither agree nor disagree” (3), “disagree” (4), and “strongly disagree” (5). After being provided with participant information sheets (PIS), students were approached and requested to sign consent forms if they agreed to participate. Additionally, students were given the opportunity to provide free-text comments at the end of the questionnaire. The 3D-printed typodont teeth were anonymised and labelled Tooth A (15 wt.% carbonated hydroxyapatite), Tooth B (15 wt.% Puraflake^®^), Tooth C (15 wt.% zinc oxide) and Tooth D (dental resin composite).

### 2.7. Open-Access Dissemination and Reproducibility

To support reproducibility and facilitate adoption by other dental schools, the digital tooth geometries, manufacturing workflows and supporting documentation developed through this programme of research have been made freely available via an institutional open-access platform (TactiTooth, Queen Mary University of London; https://www.qmul.ac.uk/dentistry/tactitooth/ (accessed on 1 September 2026) [37]). This resource provides guidance on model preparation, material selection and educational implementation to enable in-house production of haptically informed typodont teeth.

### 2.8. Statistical Analysis

Likert scale data were analysed using non-parametric statistical methods. Cutting force comparisons between each 3D-printed material and extracted enamel (Table 1) were performed using the two-sided Mann–Whitney U test, with no correction applied across the six group comparisons as these were pre-planned, single comparisons against a common reference group (extracted enamel). Differences in student perception between materials were assessed using a Friedman test, with post hoc pairwise comparisons performed using Wilcoxon signed-rank tests with Bonferroni correction. Internal consistency of the perception questionnaire was assessed using Cronbach’s alpha. The correlation between cutting force and perception score was evaluated using Spearman’s rank correlation coefficient. The statistical significance was set at *p* < 0.05.

In addition to the overall analysis, questionnaire responses were stratified by year group to examine whether perceptions differed between fourth- and fifth-year undergraduate dental students. Of the 46 respondents, 25 were fourth-year students and 21 were fifth-year students. For each typodont material, responses between year groups were compared using the Mann–Whitney U test. Given the ordinal nature of the Likert scale data, results were summarised using medians, interquartile ranges, means and standard deviations, and effect sizes were expressed as rank-biserial correlations. To determine whether the pattern of material preference was preserved within each cohort, Friedman tests were also performed separately for fourth- and fifth-year students. The statistical significance was set at *p* < 0.05.

## 3. Results

### 3.1. Haptic Response Measurements

**Figure 2:**
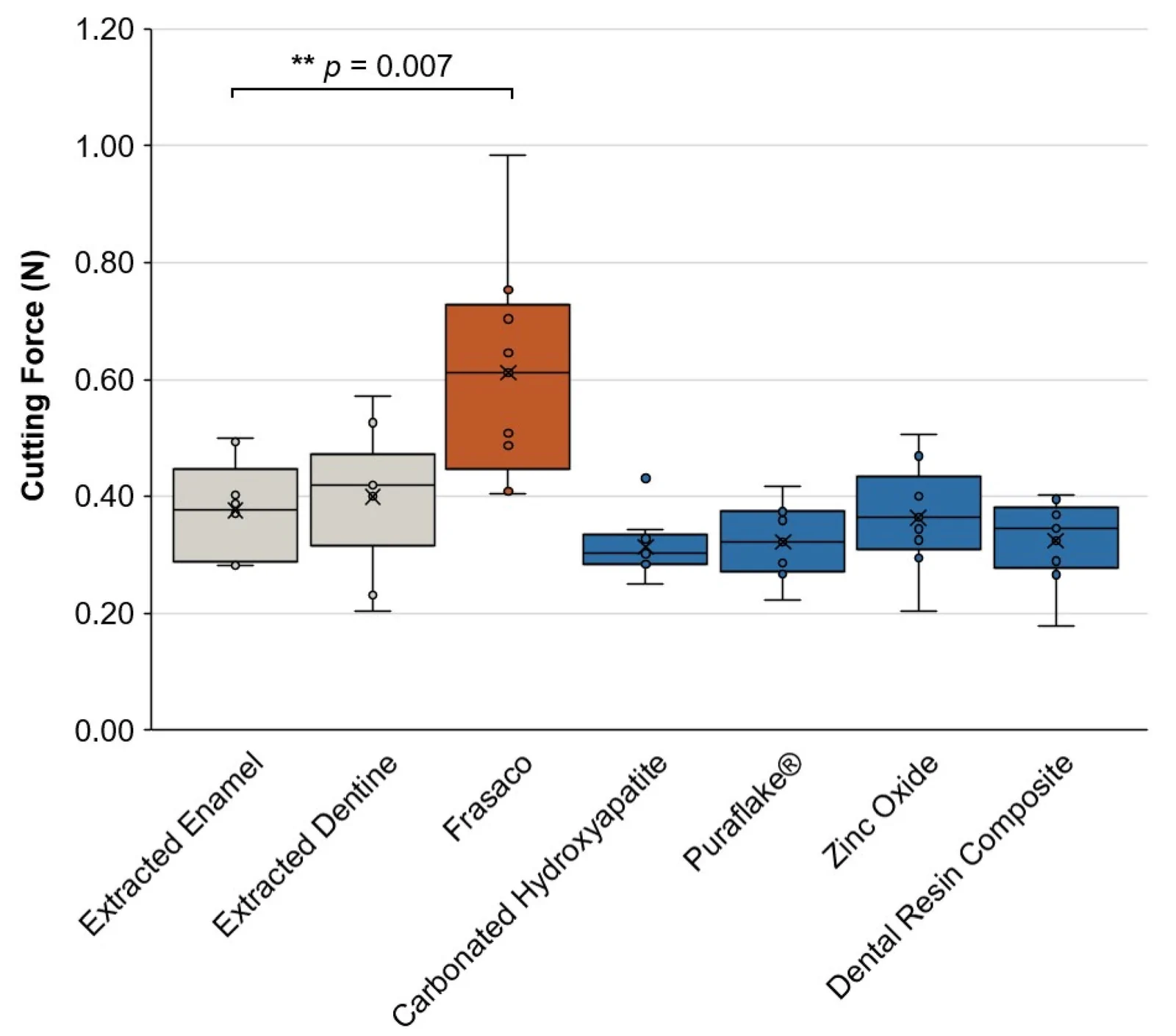
Cutting force (N) for extracted enamel, extracted dentine, Frasaco, and the four 3D-printed materials, shown as individual measurements (*n* = 8 per group) with mean ± SD. All measurements were collected as part of the present study. The Frasaco typodont required significantly greater force than extracted enamel (Mann–Whitney U = 58.0, *p* = 0.007 [**]); no other group differed significantly from extracted enamel.

The mean forces required to cut the 3D-printed typodont teeth fabricated from the four alternative materials are presented in Figure 2 and Table 1. All force measurements, including those for extracted enamel, extracted dentine, and the commercial Frasaco typodont, were collected as part of the present study’s own experiments. No statistically significant difference in cutting force was detected between any of the four 3D-printed materials and extracted enamel; all four materials sit numerically below the extracted-enamel mean, by 3–16%.

To test this more rigorously than a simple absence of significance allows, we conducted two one-sided equivalence tests (TOST) against ±10% and ±20% margins of the extracted-enamel mean. At the current sample size (*n* = 8 per group), the confidence intervals were wide enough that equivalence could not yet be formally confirmed at either margin, a precision limit rather than evidence against equivalence, and one that a larger future sample would resolve.

The mean force required to cut extracted enamel was 0.376 N (±0.088). Typodont teeth fabricated with 15 wt.% carbonated hydroxyapatite and the dental resin composite demonstrated mean cutting forces of 0.314 N (±0.055) and 0.324 N (±0.075), respectively, and typodont teeth fabricated with 15 wt.% Puraflake^®^ and 15 wt.% zinc oxide demonstrated mean cutting forces of 0.322 N (±0.068) and 0.364 N (±0.097), respectively—all below the extracted-enamel mean. A significant difference was observed between extracted enamel and the commercial Frasaco typodont, which required 62.6% more force to cut (0.612 N vs. 0.376 N; Mann–Whitney U = 58.0, *p* = 0.007).

### 3.2. Questionnaire Responses

**Figure 3:**
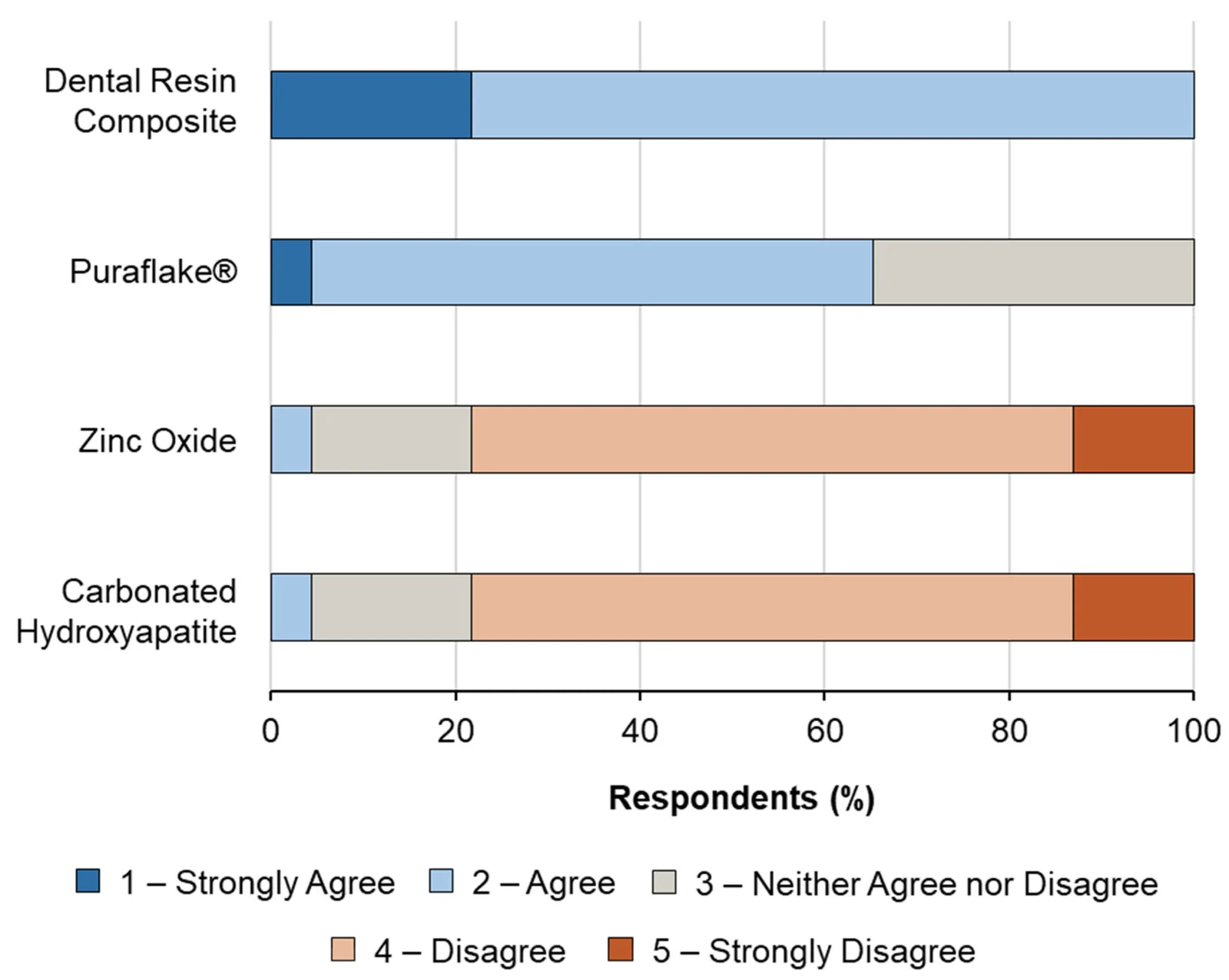
Distribution of Likert scale responses to the statement “The feel of cutting Tooth X is similar to cutting real enamel” for each of the four 3D-printed typodont materials (*n* = 46). Materials are ordered from most to least favourably rated. The dental resin composite received exclusively favourable responses (strongly agree or agree), whereas the carbonated hydroxyapatite and zinc oxide composites were rated predominantly unfavourably, despite no statistically significant difference in cutting force being detected between any of the four materials and extracted enamel.

Of the 83 undergraduate dental students approached, 46 completed questionnaires were returned, representing a participation rate of 55.42%. Participants spent approximately 5–15 min interacting with the 3D-printed typodont teeth prior to completing the questionnaire. Figure 3 and Table 2 summarise the Likert scale responses.

Overall, the dental resin composite typodont teeth received the most favourable ratings, with a mean score of 1.72 (±0.46) (lower scores indicate greater perceived similarity to extracted enamel; Table 2). This preference was reflected in item 4 of the questionnaire, with which 28.26% of respondents strongly agreed and 71.74% agreed that the dental composite resin felt similar to extracted enamel. Typodont teeth fabricated with 15 wt.% carbonated hydroxyapatite received the lowest ratings, with a mean score of 3.96 (±0.76), despite exhibiting cutting forces comparable to extracted enamel.

In item 5, all 46 respondents (100%) indicated they would use the 3D-printed typodont teeth in their training again. In item 6 (tick all that apply), the dental resin composite was selected by 38 of 46 respondents (82.6%) and Puraflake^®^ by 8 of 46 respondents (17.4%); neither the zinc oxide nor carbonated hydroxyapatite typodont was selected by any respondent. This closed-question preference for the dental resin composite is consistent with its favourable Likert scale ratings reported above and with its status as the material producing the lowest post-cut surface roughness relative to extracted enamel.

### 3.3. Statistical Analysis of Questionnaire Responses

Likert scale questionnaire data were analysed using non-parametric statistical methods. A Friedman test revealed a statistically significant difference in student perception between the four 3D-printed typodont materials (χ^2^ (3) = 123.96, *p* < 0.001) (Figure 3). The effect size, assessed using Kendall’s coefficient of concordance, indicated a very large effect (W = 0.90), demonstrating a high level of agreement among participants in the ranking of materials.

Post hoc Wilcoxon signed-rank tests with Bonferroni correction indicated that the dental composite resin was rated significantly more favourably than the carbonated hydroxyapatite, Puraflake^®^ and zinc oxide composites (all *p* < 0.001). Puraflake^®^ was also rated significantly more favourably than the carbonated hydroxyapatite composite (*p* < 0.001). The perception questionnaire’s four items showed a Cronbach’s alpha of 0.81; however, as each item refers to a different material, this study shows that they differ significantly in perception score. We present this value only as a descriptive measure of respondent-level response tendency, noting that all four items are worded in the same direction with no reverse-scored item. No significant correlation was observed between the mean cutting force and mean perception score across materials (Spearman’s ρ = −0.40, *p* = 0.60); at *n* = 4 material means, no Spearman correlation can reach *p* < 0.05 (minimum attainable two-sided *p* = 0.083), highlighting the limited resolution of material-level comparisons at this sample size and motivating the respondent-level analysis presented below (Page’s L trend test), which achieves substantially greater statistical power for testing this relationship.

**Figure 4:**
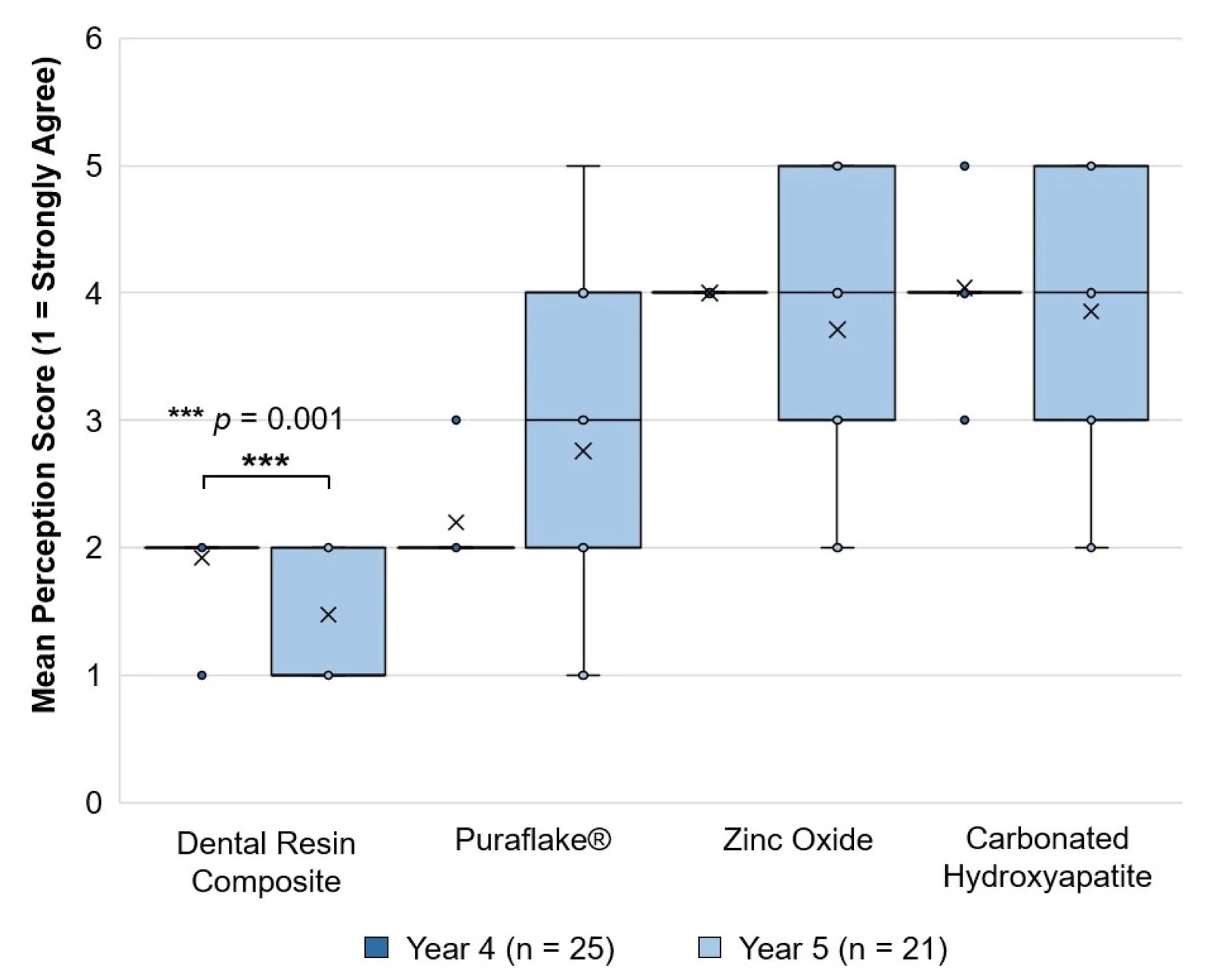
Likert scale perception scores by material and year group, shown as median, interquartile range, and individual responses (Year 4 *n* = 25, Year 5 *n* = 21). Only the dental resin composite difference remained significant after Bonferroni correction for four comparisons (adjusted α = 0.0125; *p* = 0.001 [***]). All 25 Year 4 responses for zinc oxide were identical (rating of 4), confirmed against the raw data as a genuine consensus rather than a data-handling artefact.

Of these respondents, 25 were in Year 4 and 21 were in Year 5. The cohort-stratified analysis demonstrated that the overall ranking of materials was preserved in both year groups, with Friedman tests indicating a statistically significant difference between the four materials in Year 4 (χ^2^ (3) = 71.46, *p* < 0.001) and in Year 5 (χ^2^ (3) = 56.51, *p* < 0.001) (Figure 4).

When responses were compared between year groups for each material, the most pronounced between-cohort difference was observed for the dental resin composite. Year 5 dental students rated the dental resin-based composite more favourably than Year 4 students (Year 4 mean 1.92 ± 0.28; Year 5 mean 1.48 ± 0.51; Mann–Whitney U = 379.0, *p* = 0.001; rank-biserial correlation = −0.44). An uncorrected difference was also observed for Puraflake^®^ (*p* = 0.045), with fourth-year students rating it more favourably; however, this does not survive Bonferroni correction (see Table 3) and is not presented here as a reliable effect. No significant differences were observed for carbonated hydroxyapatite (*p* = 0.632) or zinc oxide (*p* = 0.197) (Table 3).

These findings suggest that both cohorts discriminated strongly between the tested materials, but that fifth-year students showed a stronger preference for the dental resin composite than fourth-year students.

### 3.4. Surface Roughness Measurements

**Figure 5:**
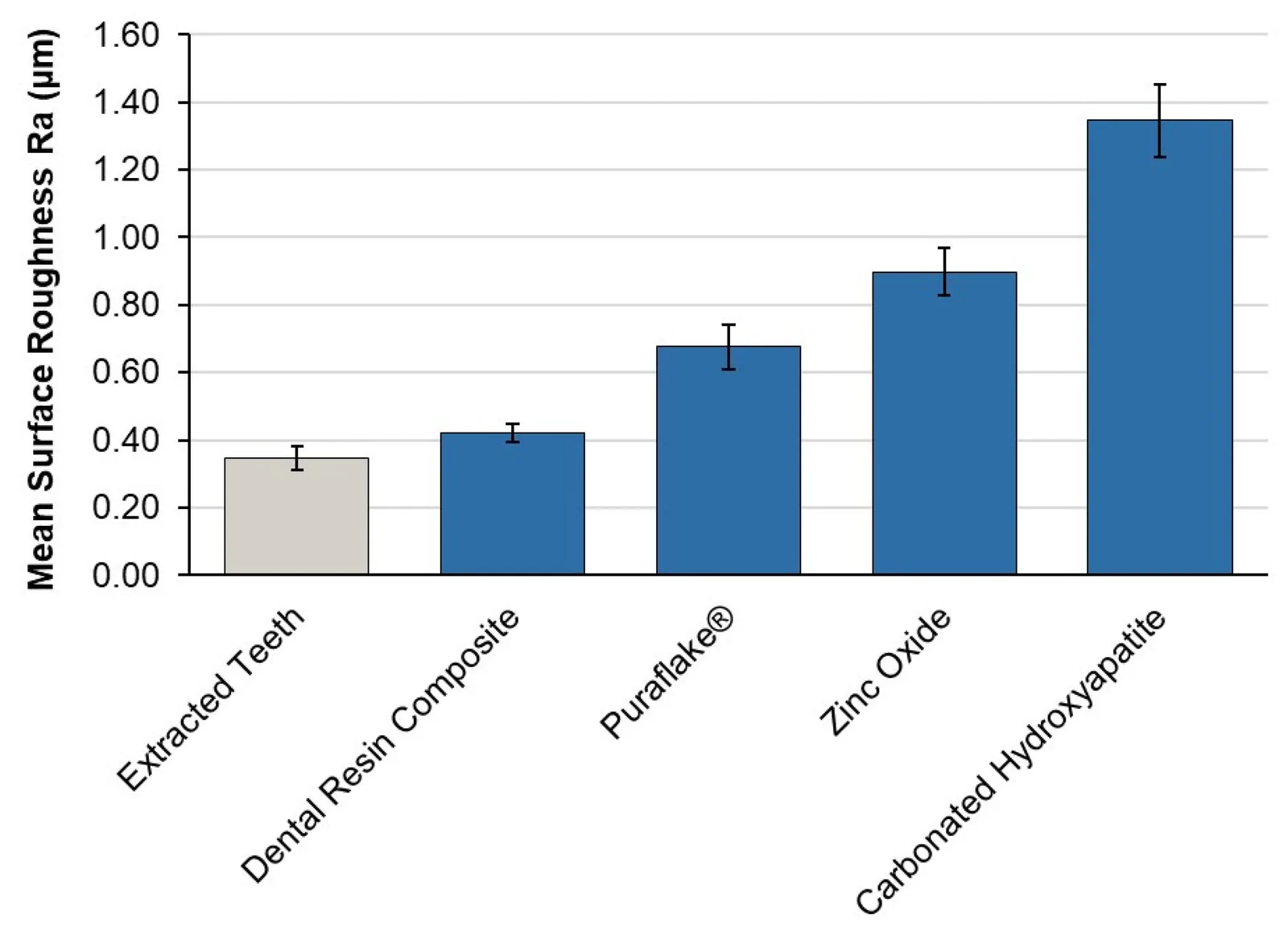
Mean post-cut surface roughness (Ra, μm) for extracted teeth and the four 3D-printed typodont materials, measured under identical cutting conditions. Error bars represent ±1 standard deviation (*n* = 8 per group). Materials are ordered from lowest to highest Ra. The dental resin composite produced the smoothest post-cut surface and most closely approximated the Ra of extracted teeth, while the carbonated hydroxyapatite composite produced the roughest surface.

Mean post-cut surface roughness (Ra) values for extracted teeth and the four 3D-printed typodont materials are presented in Figure 5. Extracted teeth exhibited a mean Ra of 0.35 µm (±0.04), providing the reference surface texture against which the printed materials were compared. Among the 3D-printed materials, the dental resin composite produced the smoothest post-cut surface (Ra 0.42 µm ± 0.03), most closely approximating the texture of extracted teeth. The Puraflake^®^ composite produced an intermediate surface roughness (Ra 0.68 µm ± 0.07), followed by the zinc oxide composite (Ra 0.90 µm ± 0.07). Carbonated hydroxyapatite composite produced the roughest post-cut surface (Ra 1.35 µm ± 0.11), representing an approximately four-fold increase relative to extracted teeth.

**Figure 6:**
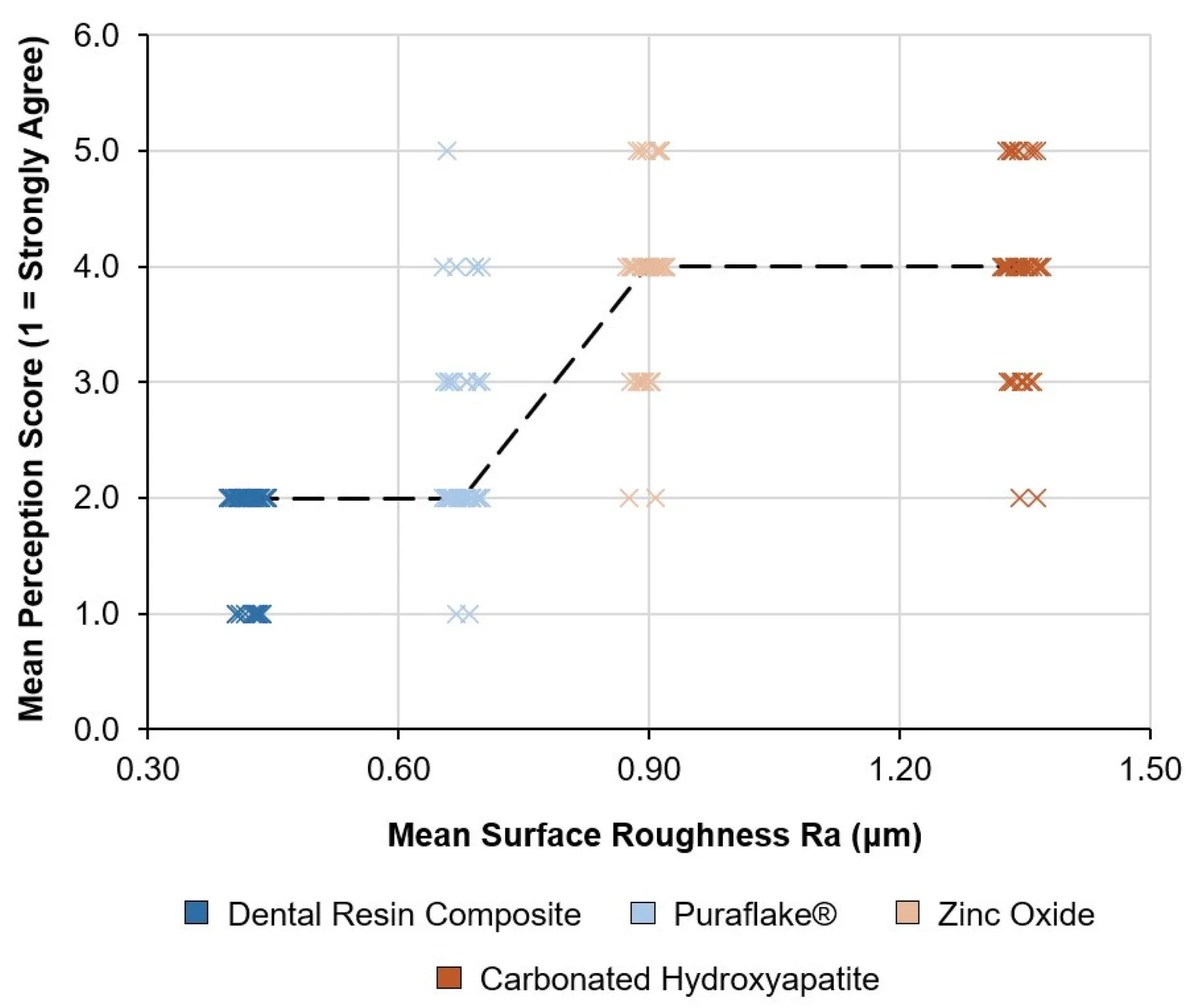
Individual respondent perception scores (*n* = 46 students × 4 materials) plotted against each material’s mean post-cut surface roughness (Ra, μm), with points jittered horizontally for visibility. The dashed line connects each material’s median perception score. A strong, statistically robust monotonic trend was confirmed using Page’s L trend test on the individual respondent ratings (L = 1339.5, *p* = 1.86 × 10^−22^), with materials ordered by ascending Ra as the a priori test sequence. Lower scores indicate greater perceived similarity to extracted enamel. Note that carbonated hydroxyapatite’s roughness (1.35 μm) is disproportionately higher than that of the other three materials, which cluster more closely together (0.42–0.90 μm).

The rank ordering of materials by Ra corresponded exactly to the rank ordering by mean perception score (Figure 6). Because a Spearman correlation across only four material means cannot validly reach *p* < 0.001 (minimum attainable two-sided *p* = 0.083 at *n* = 4), we instead tested this relationship at the individual-respondent level using Page’s L trend test, treating the four materials as an a priori ordered sequence (by ascending Ra) across all 46 respondents’ ratings. This revealed a strong, statistically robust monotonic association (L = 1339.5, *p* = 1.86 × 10^−22^; a sanity check with materials in reversed Ra order returns *p* = 1.0, confirming the direction of the effect). No significant correlation was observed between cutting force and perception score (ρ = −0.40, *p* = 0.60) (Supplementary Figure S2).

### 3.5. Free-Text Responses

Approximately four respondents (8% of 46) provided free-text comments. Given this small number, we present a brief summary rather than a thematic analysis: comments broadly corroborated the quantitative findings, most frequently noting the dental resin composite as feeling most realistic and the printed typodonts generally as being easier to control than the commercial comparator.

## 4. Discussion

The dissociation between cutting force and perceived realism observed here indicates that force-matching, while necessary, characterises only one dimension of the cutting experience. Where force differences were statistically undetectable, students nonetheless discriminated sharply and consistently between materials, a pattern that points toward surface-level cues generated during material removal, rather than the mean applied force, as a more immediate driver of perceived similarity to natural enamel.

Cutting force measurements found no statistically significant difference between any of the four 3D-printed typodont materials and extracted enamel. This is further supported by formal TOST equivalence testing, which points to the sample size needed for this equivalence to be established with full statistical confidence in future work. This is consistent with previous work demonstrating that composite materials can be engineered to replicate dental tissue cutting resistance when appropriate relative mechanical properties are achieved [31,32,33]. In contrast, commercial typodont teeth required substantially greater force to cut, supporting earlier reports that conventional polymer-based typodonts provide an operative experience that differs markedly from natural teeth [1,8,9].

Despite the absence of a detectable difference in measured cutting force, the Friedman test demonstrated a highly significant difference in student perception between materials (χ^2^ (3) = 123.96, *p* < 0.001), with a very large effect size (Kendall’s W = 0.90). This indicates not only that perceptual differences existed, but that there was strong agreement among participants in how the materials were ranked. Post hoc analysis demonstrated that the dental resin composite was rated significantly more favourably than all other materials, while the carbonated hydroxyapatite composite was rated significantly lower. This pattern was corroborated by an independent, closed-question measure: when asked which typodont they would be willing to use again (item 6), respondents selected only the dental resin composite (38 of 46) and Puraflake^®^ (8 of 46); no respondent selected the zinc oxide or carbonated hydroxyapatite typodont, despite all four materials having been cut by every participant. The convergence of the Likert scale ratings, the Friedman/post hoc analysis, and this independent binary preference measure indicates that force similarity alone did not translate into perceptual similarity for this cohort.

The cohort-stratified analysis provides further insight into how learner experience may influence perceptual judgements of haptic realism. After applying a Bonferroni correction across the four material-wise year-group comparisons (adjusted α = 0.0125), only the dental resin composite difference remained statistically robust: fifth-year students rated it significantly more favourably than fourth-year students (*p* = 0.001). An uncorrected difference was also observed for Puraflake^®^ (*p* = 0.045), but this did not survive correction. One plausible explanation for the composite finding is that fifth-year students, having accumulated more clinical experience cutting extracted enamel, may be better calibrated to detect the closer haptic match this material offers; however, since no extracted-tooth reference was provided during the perception session itself, students’ judgements necessarily depended on recalled experience, which is itself likely to differ systematically between cohorts. This confound limits how strongly the year-group finding can be attributed to genuine perceptual sensitivity rather than differences in recall. No statistically significant year-group differences were observed for carbonated hydroxyapatite or zinc oxide.

The perception questionnaire’s four items showed a Cronbach’s alpha of 0.81. However, as each item refers to a different material, and this study itself demonstrates that these materials differ significantly in perceived similarity to enamel, the four items cannot be assumed to measure a single latent construct; alpha in this context is better interpreted as a measure of respondents’ consistency in response direction (acquiescence) rather than as validity evidence for the underlying perceptual data.

No significant correlation was observed between the mean cutting force and mean perception score across materials (Spearman’s ρ = −0.40, *p* = 0.60); this correlation is based on only four material-level data points, and no Spearman correlation at *n* = 4 can reach *p* < 0.05, so the null result should not be read as evidence that force and perception are unrelated. This likely also reflects the narrow range of material-mean cutting forces tested here (0.314–0.364 N), as the four materials were deliberately selected to cluster close to extracted enamel; across a wider range of typodont materials, including commercial products with cutting forces several-fold higher than extracted enamel, cutting force has previously been shown to relate strongly to material mechanical properties and to perceived likeness to extracted teeth [31,32,33]. Instead, once cutting force is already closely matched, as it was here, finer perceptual differences between materials appear to be driven more by surface behaviour during cutting than by residual differences in mean force.

This aligns with evidence from machining and haptics research demonstrating that perceived resistance is influenced by dynamic force fluctuations, vibration and acoustic feedback rather than static force alone [27,28,29]. The quantitative differences in post-cut surface roughness observed between materials (Figure 5) and the strong, respondent-level association between Ra and perception score together suggest that material removal behaviour, rather than mean cutting force, drives perceived realism. Materials producing higher Ra values, such as carbonated hydroxyapatite, may fracture in a more brittle, irregular manner, generating higher-frequency force transients and surface irregularities that are perceptible to the operator even when mean forces are equivalent. We present this explicitly as a hypothesis requiring direct force-time measurement (e.g., coefficient of variation during cutting) to confirm, rather than as an established mechanism. The dental resin composite, which most closely approximated the post-cut surface texture of extracted teeth (Ra 0.42 µm vs. 0.35 µm), was also rated most favourably by students, while the carbonated hydroxyapatite composite, which produced the roughest surface (Ra 1.35 µm), received the lowest perception scores.

The discrepancy between objective and subjective outcomes may also reflect differences between novice and expert operators. Undergraduate students may prioritise surface-level cues and consistency of resistance, whereas experienced clinicians integrate tactile feedback over longer time scales and depths [30,32]. Previous evaluations involving dental clinicians reported favourable cutting characteristics for force-matched 3D-printed typodont teeth [32], suggesting that perceptual priorities differ between learner groups.

The strong, statistically robust monotonic association observed between post-cut surface roughness and perception score at the respondent level (Page’s L trend test, *n* = 46, *p* < 0.001; see Results) is a notable finding. Because it is derived from individual ratings across all 46 respondents rather than four material-level averages, it offers considerably more confidence than a material-mean correlation could, though it remains an association rather than a demonstration of a causal mechanism. The direction and consistency of the finding are compelling: across all four materials, the rank order of Ra values corresponded exactly to the rank order of perceived similarity to extracted enamel. This suggests that surface behaviour during cutting is at least as informative as, and possibly more discriminating than, mean cutting force as a determinant of perceived haptic realism for undergraduate learners. We present this as a robust pattern warranting confirmatory investigation with direct force-time and acoustic measurement, rather than as a full demonstration of the underlying mechanism. Future work incorporating a wider range of materials with deliberately varied Ra profiles would allow this relationship to be tested more rigorously.

Surface roughness may act as a proxy for broader material removal characteristics including fracture mode, chip formation and elastic recovery, any of which could contribute independently to perceived realism. 

Taken together, these findings support evaluating typodont materials on multiple criteria: mechanical measurement, perceptual data, and material behaviour during cutting, rather than cutting force alone. Similar limitations have been noted in studies of anatomically accurate 3D-printed teeth, where high geometric fidelity was achieved but material behaviour remained a recognised constraint [12,13].

The open-access dissemination of the digital models and manufacturing workflows via the TactiTooth platform addresses an additional limitation identified in the literature, namely the lack of reproducibility and accessibility of novel typodont designs [16,18]. By enabling in-house fabrication and iterative refinement informed by educational feedback, this approach supports wider adoption and future optimisation. 

This study presented several limitations. The typodont teeth were based on a single tooth geometry (a mandibular first molar), which may not generalise to other tooth types or preparation contexts. The cutting-force comparison relies on a modest per-group sample size (*n* = 8), which provided sufficient power to detect large differences (e.g., versus the commercial Frasaco typodont) but was not sufficient to formally establish statistical equivalence between the printed materials and extracted enamel via two one-sided testing; the reported absence of a significant difference should be interpreted accordingly. The perception questionnaire did not provide students with a concurrent extracted-tooth reference during testing, so responses depended on recalled experience of cutting enamel, which may differ systematically between year groups and represents a direct confound for the year-group comparison reported here. Although students were not informed of material composition at any point, the four typodont teeth remain visually distinguishable from one another (Figure 1); we cannot rule out that this visual impression, independent of any knowledge of composition, contributed to perceived dissimilarity from extracted enamel for some materials. Sedimentation of the 15 wt.% zinc oxide filler suspension during printing was assumed to be negligible rather than empirically verified. Exposure time to the typodont teeth during the perception task was relatively short (5–15 min) and varied between participants, and the free-text response rate was modest (*n* ≈ 4, 8%), limiting the depth of qualitative insight available. Finally, while the respondent-level roughness-perception association reported here is statistically robust, it remains an association derived from four discrete material formulations rather than a continuously varied roughness manipulation, and the proposed brittle-fracture/force-transient mechanism remains a hypothesis requiring direct force-time and acoustic measurement to confirm. Future work should prioritise this confirmatory testing, alongside evaluation across a broader range of materials and assessment of longer-term educational outcomes.

## 5. Conclusions

This study demonstrates that 3D-printed typodont teeth fabricated from alternative composite materials can be engineered to exhibit mean cutting forces statistically indistinguishable from extracted enamel, though the study was not powered to formally establish equivalence. Force similarity alone did not ensure perceptual similarity for undergraduate dental students. Among the materials tested, the dental resin composite received the most favourable and most consistent perception scores and produced the post-cut surface roughness closest to extracted teeth; based on the totality of evidence presented here, we recommend the dental resin composite as the preferred material for pre-clinical typodont fabrication among those tested, with the roughness-matched alternatives available as viable options where dental resin composite fabrication is not accessible. Across the four printed materials, post-cut surface roughness showed a strong, respondent-level association with perceived haptic similarity. We prioritise direct measurement of dynamic cutting cues (force fluctuations, vibration and acoustic feedback) as the most immediate future research direction, followed by evaluation across a broader range of materials and longer-term educational outcomes.

## Figures and Tables

**Table 1:** Summary of cutting force measurements for all materials. Values are presented as mean and standard deviation (SD) with the observed range across eight repeat measurements. Statistical significance was assessed using the Mann–Whitney U test (two-sided) with extracted enamel as the reference group. ns = not significant (*p* > 0.05).

Material	Mean (N)	SD	Range	vs. Enamel
Extracted enamel	0.376	0.088	0.282–0.500	—
Extracted dentine	0.400	0.128	0.203–0.571	ns
Frasaco (commercial)	0.612	0.199	0.405–0.983	*p* = 0.007
Carbonated Hydroxyapatite	0.314	0.055	0.251–0.431	ns
Puraflake^®^	0.322	0.068	0.223–0.418	ns
Zinc Oxide	0.364	0.097	0.203–0.507	ns
Dental Resin	0.324	0.075	0.179–0.402	ns

**Table 2:** Perception summary statistics and post hoc pairwise comparisons for the four 3D-printed typodont materials. Likert scale data are presented as mean, standard deviation (SD), median, and interquartile range (IQR) for all 46 respondents (1 = strongly agree that the feel is similar to extracted enamel, 5 = strongly disagree). Overall group differences were assessed using the Friedman test (χ^2^ (3) = 123.96, *p* < 0.001, Kendall’s W = 0.90). Post hoc pairwise comparisons were performed using the Wilcoxon signed-rank test with Bonferroni correction (six comparisons; adjusted significance threshold *p* < 0.0083). W = Wilcoxon test statistic. ns = not significant. *** *p* < 0.001.

Material	Mean	SD	Median	IQR
Dental Resin Composite	1.72	0.46	2.0	1.0–2.0
Puraflake^®^	2.46	0.84	2.0	2.0–3.0
Zinc Oxide	3.87	0.69	4.0	4.0–4.0
Carbonated Hydroxyapatite	3.96	0.76	4.0	4.0–4.0
Post hoc pairwise comparisons (Wilcoxon signed-rank, Bonferroni corrected)				
**Comparison**	**W**	**Non-zero pairs (of 46)**	***p* (Bonferroni-adjusted)**	**Sig.**
Dental Resin Composite vs. Puraflake^®^	0	26	1.79 × 10^−7^	***
Dental Resin Composite vs. Zinc Oxide	0	46	1.70 × 10^−13^	***
Dental Resin Composite vs. Carbonated Hydroxyapatite	0	46	1.70 × 10^−13^	***
Puraflake^®^ vs. Zinc Oxide	0	40	1.09 × 10^−11^	***
Puraflake^®^ vs. Carbonated Hydroxyapatite	0	40	1.09 × 10^−11^	***
Zinc Oxide vs. Carbonated Hydroxyapatite	7	7	1.000	ns

**Table 3:** Mann–Whitney U test results comparing Likert scale perception scores between Year 4 (*n* = 25) and Year 5 (*n* = 21) undergraduate dental students for each of the four 3D-printed typodont materials. Values are presented as mean ± standard deviation. Rank-biserial correlation (r) is reported as a measure of effect size. Lower scores indicate greater perceived similarity to extracted enamel. Bold *p*-values indicate statistical significance (*p* < 0.05). At a Bonferroni-adjusted threshold of 0.0125 (four comparisons), only the dental resin composite difference survives correction; the Puraflake^®^ difference (*p* = 0.045) does not.

Material	Year 4 Mean ± SD	Year 5 Mean ± SD	U	*p*	Rank-Biserial r
Dental Resin Composite	1.92 ± 0.28	1.48 ± 0.51	379.0	**0.001**	−0.44
Puraflake^®^	2.20 ± 0.41	2.76 ± 1.09	182.5	**0.045**	0.30
Zinc Oxide	4.00 ± 0.00	3.71 ± 1.01	312.5	0.197	−0.19
Carbonated Hydroxyapatite	4.04 ± 0.45	3.86 ± 1.01	282.5	0.632	−0.08

## Data Availability

The original contributions presented in the study are included in the article/Supplementary Material, further inquiries can be directed to the corresponding author.

## Supplementary Materials

The following supporting information can be downloaded at https://www.mdpi.com/article/10.3390/jfb17090471/s1. Figure S1: Likert scale questionnaire administered to fourth- and fifth-year undergraduate dental students. Section A comprised four perception-based questions rated on a five-point Likert scale (1 = strongly agree, 5 = strongly disagree). Section B comprised two closed-ended questions assessing willingness to use the typodont teeth in future training. Tooth identities were anonymised during data collection. Tooth A = 15 wt.% carbonated hydroxyapatite composite; Tooth B = 15 wt.% Puraflake^®^ composite; Tooth C = 15 wt.% zinc oxide composite; Tooth D = dental resin composite. Material identities were not disclosed to participants prior to or during questionnaire completion; Figure S2: Relationship between mean cutting force (N) and mean Likert scale perception score for the four 3D-printed typodont materials. Each point represents one material (*n* = 8 cutting force measurements; *n* = 46 perception ratings per material). Error bars represent ±1 standard deviation on both axes. No significant correlation was observed between cutting force and perception score (Spearman ρ = −0.40, *p* = 0.60), indicating that objective force equivalence does not predict perceived haptic similarity to extracted enamel. Lower perception scores indicate greater perceived similarity to enamel; Table S1: Material characterisation summary for the four 3D-printed typodont formulations.

## Abbreviations

The following abbreviations are used in this manuscript:

CHAp	Carbonated hydroxyapatite
UDMA	Urethane dimethacrylate
TEGDMA	Triethylene glycol dimethacrylate
Ra	Surface roughness
SD	Standard deviation
IQR	Interquartile range

## References

[1] Al-Sudani D.I., Basudan S.O. (2017). Students’ perceptions of pre-clinical endodontic training with artificial teeth compared to extracted human teeth. Eur. J. Dent. Educ..

[2] Lone M., McKenna J.P., Cryan J.F., Downer E.J., Toulouse A. (2018). A Survey of tooth morphology teaching methods employed in the United Kingdom and Ireland. Eur. J. Dent. Educ..

[3] Brand H.S., van der Cammen C.C.J., Roorda S.M.E., Baart J.A. (2015). Tooth extraction education at dental schools across Europe. BDJ Open.

[4] Decurcio D.A., Lim E., Nagendrababu V., Estrela C., Rossi-Fedele G. (2020). Difficulty levels of extracted human teeth used for pre-clinical training in endodontics in an Australian dental school. Aust. Endod. J..

[5] Holden A.C.L., Dracopoulos S.A. (2017). Owning the tooth: Exploring the ethical and legal issues relating to the use of extracted human teeth in dental education in Australia. Aust. Dent. J..

[6] Deogade S.C., Mantri S.S., Saxena S., Sumathi K. (2016). Awareness and Knowledge of Undergraduate Dental Students about Sterilization/Disinfection Methods of Extracted Human Teeth. Ann. Med. Health Sci. Res..

[7] Bitter K., Gruner D., Wolf O., Schwendicke F. (2016). Artificial Versus Natural Teeth for Preclinical Endodontic Training: A Randomized Controlled Trial. J. Endod..

[8] Lee B., Kim J.-E., Shin S.-H., Kim J.-H., Park J.-M., Kim K.-Y., Kim S.-Y., Shim J.-S. (2022). Dental students’ perceptions on a simulated practice using patient-based customised typodonts during the transition from preclinical to clinical education. Eur. J. Dent. Educ..

[9] Park K., Asnashari K., Younan R., Becker R., Roggenkamp C., Oyoyo U., Kwon S.R. (2024). Evaluating a method of creating artificial caries in typodont teeth for teaching Class III cavity preparations. J. Dent. Educ..

[10] Kang A., Firth F.A., Antoun J., Mei L., Ali A., Farella M. (2023). Three-dimensional digital assessment of typodont activations. Orthod. Craniofac. Res..

[11] Goldschmidt S.L., Root Kustritz M.V. (2021). Pilot Study Evaluating the Use of Typodonts (Dental Models) for Teaching Veterinary Dentistry as Part of the Core Veterinary Curriculum. J. Vet. Med. Educ..

[12] Höhne C., Schmitter M. (2019). 3D Printed Teeth for the Preclinical Education of Dental Students. J. Dent. Educ..

[13] Höhne C., Schwarzbauer R., Schmitter M. (2019). 3D Printed Teeth with Enamel and Dentin Layer for Educating Dental Students in Crown Preparation. J. Dent. Educ..

[14] Cresswell-Boyes A.J., Barber A.H., Mills D., Tatla A., Davis G.R. (2018). Approaches to 3D printing teeth from X-ray microtomography. J. Microsc..

[15] Ramírez-Muñoz A., Escribano-Capdevila M., Navarrete N., Vieira G.C.S., Salamanca-Ramos M., Ortolani-Seltenerich P.S., Aranguren J., Pérez A.R. (2024). Comparative Micro-CT Analysis of Minimally Invasive Endodontic Systems Using 3D-Printed Replicas and Natural Teeth. Materials.

[16] Göksu M., Tosun S., Ertuğrul I.F. (2024). Evaluation of the Use of 3D-Printed Tooth Models in Endodontic Practical Training. Lokman Hekim Health Sci..

[17] Suresh Kumar B., Baskar N., Rajaguru K. (2020). Drilling operation: A review. Mater. Today Proc..

[18] Zhao J., Wu D., Liu S., Zhang Z., Zhao J. (2023). Enamel cutting mechanism and performance of different dental burs: An in vitro study. Mater. Res. Express.

[19] Wu S.-X., Li K.-Q., Zhu W.-Z., Wang C.-Y., Chen W.-L. (2020). Machinability of high-speed enamel cutting with carbide bur. J. Mech. Behav. Biomed. Mater..

[20] Lee B., Hwang J., Lim J.-H., Kim J.-E., Shim J.-S., Shin Y. (2024). Three-axis load analysis of high-speed handpiece on dental training teeth and computer-aided design/computer-aided manufacturing blocks. J. Mech. Behav. Biomed. Mater..

[21] Wang J., Zhang J., Feng P., Guo P. (2018). Experimental and theoretical investigation on critical cutting force in rotary ultrasonic drilling of brittle materials and composites. Int. J. Mech. Sci..

[22] Mishnaevsky L.L. (1994). Investigation of the cutting of brittle materials. Int. J. Mach. Tools Manuf..

[23] Liu K., Li X.P., Liang S.Y. (2007). The mechanism of ductile chip formation in cutting of brittle materials. Int. J. Adv. Manuf. Technol..

[24] Lei Y., Gao J., Xu Z., Guo B., Wang M., Zhong W., Zeng W., Xu X. (2025). Study on the formation mechanism and morphology of edge burrs in radial fly-cutting of triangular pyramid microstructures. J. Mater. Res. Technol..

[25] Tsao C.C., Hocheng H. (2008). Evaluation of thrust force and surface roughness in drilling composite material using Taguchi analysis and neural network. J. Mater. Process. Technol..

[26] Lin T.R. (2002). Cutting Behaviour Using Variable Feed and Variable Speed when Drilling Stainless Steel with TiN-Coated Carbide Drills. Int. J. Adv. Manuf. Technol..

[27] Priest J., Ghadbeigi H., Avar-Soberanis S., Gerardis S. (2021). 3D finite element modelling of drilling: The effect of modelling method. CIRP J. Manuf. Sci. Technol..

[28] Jarosz K., Zhang Y., Liu R. Investigating the Role of Auditory Perception of Cutting Process Conditions in CNC Machining. Proceedings of the ASME 2022 17th International Manufacturing Science and Engineering Conference.

[29] Kim J.-B., Lee S.-J., Park Y.-P. (1994). Stable and efficient drilling process by active control of the thrust force. Mech. Syst. Signal Process..

[30] Patil S., Bhandi S., Awan K.H., Licari F.W., Di Blasio M., Ronsivalle V., Cicciù M., Minervini G. (2023). Effectiveness of haptic feedback devices in preclinical training of dental students-a systematic review. BMC Oral Health.

[31] Cresswell-Boyes A.J., Davis G.R., Krishnamoorthy M., Mills D., Barber A.H. (2022). Composite 3D printing of biomimetic human teeth. Sci. Rep..

[32] Cresswell-Boyes A.J., Davis G.R., Barber A.H., Krishnamoorthy M., Nehete S.R. (2025). An evaluation by dental clinicians of cutting characteristics and haptic perceptions in 3D-printed typodont teeth: A pilot study. J. Dent. Educ..

[33] Cresswell-Boyes A.J., Davis G.R., Baysan A. (2024). Students’ perceptions of endodontic typodont teeth with simulated canals printed from novel materials. Front. Dent. Med..

[34] Davis G.R., Evershed A.N.Z., Mills D. (2013). Quantitative high contrast X-ray microtomography for dental research. J. Dent..

[35] Landi E., Celotti G., Logroscino G., Tampieri A. (2003). Carbonated hydroxyapatite as bone substitute. J. Eur. Ceram. Soc..

[36] Liu S., Li K.W., Chen S.G., Yang J.Z., Jia Y.G., Wang L., Ren L. (2019). 3D printing dental composite resins with sustaining antibacterial ability. J. Mater. Sci..

[37] Queen Mary University of London TactiTooth. https://www.qmul.ac.uk/dentistry/tactitooth/.

