# Supplementary material for: Perceptual Evaluation of 3D-Printed Typodont Teeth with Comparable Cutting Forces
Source: J Funct Biomater. 2026 Sep 17;17(9):471. doi: 10.3390/jfb17090471 (PMC13608044; doi:10.3390/jfb17090471)
Supplement: Supplementary file 1 [file jfb-17-00471-s001.zip › jfb-4487749-supplementary.pdf]

Supplementary Materials

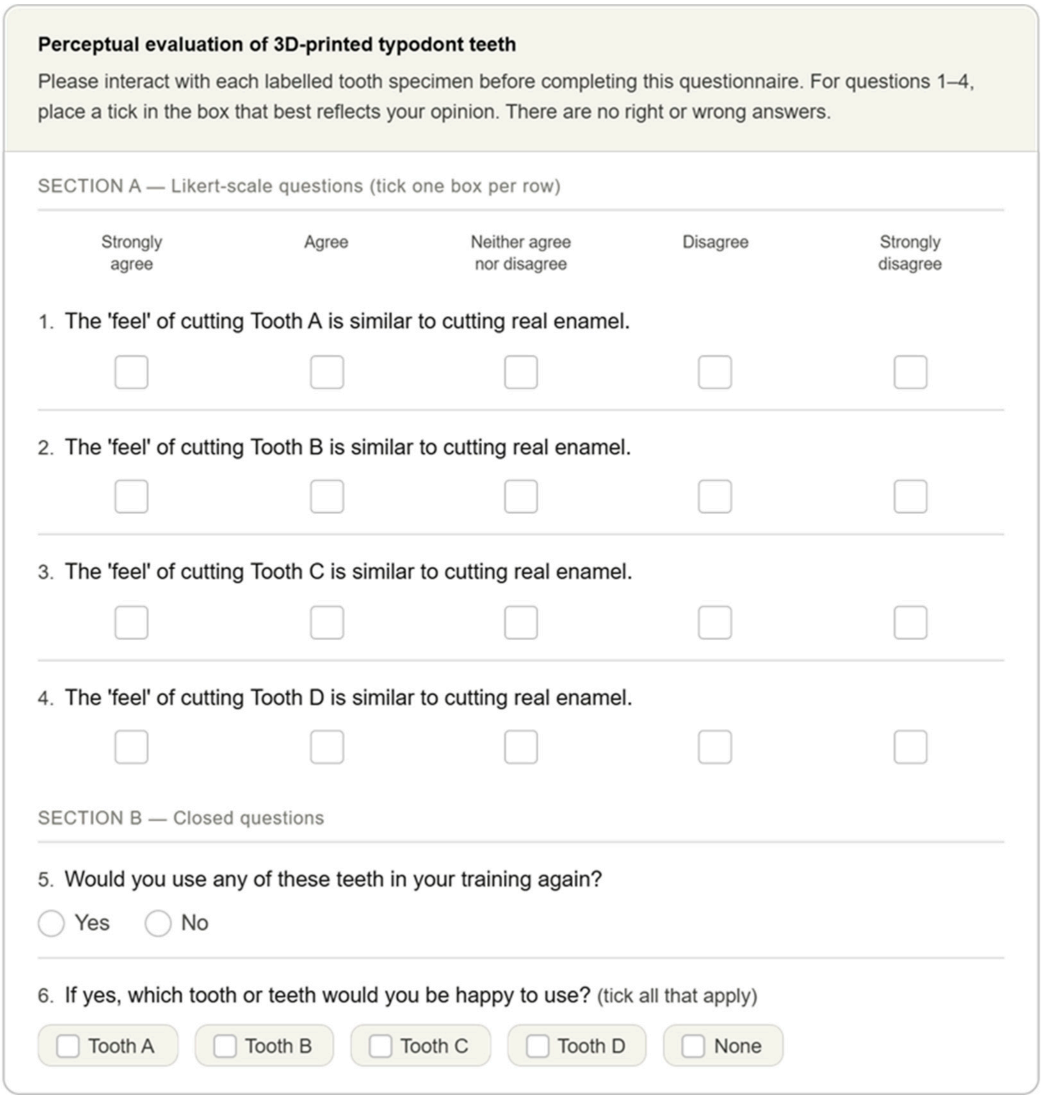

Perceptual evaluation of 3D-printed typodont teeth

Please interact with each labelled tooth specimen before completing this questionnaire. For questions 1–4, place a tick in the box that best reflects your opinion. There are no right or wrong answers.

SECTION A — Likert-scale questions (tick one box per row)

	Strongly agree	Agree	Neither agree nor disagree	Disagree	Strongly disagree
1. The 'feel' of cutting Tooth A is similar to cutting real enamel.	☐	☐	☐	☐	☐
2. The 'feel' of cutting Tooth B is similar to cutting real enamel.	☐	☐	☐	☐	☐
3. The 'feel' of cutting Tooth C is similar to cutting real enamel.	☐	☐	☐	☐	☐
4. The 'feel' of cutting Tooth D is similar to cutting real enamel.	☐	☐	☐	☐	☐

SECTION B — Closed questions

5. Would you use any of these teeth in your training again?

◯ Yes ◯ No

6. If yes, which tooth or teeth would you be happy to use? (tick all that apply)

☐ Tooth A ☐ Tooth B ☐ Tooth C ☐ Tooth D ☐ None

Figure S1. Likert-scale questionnaire administered to fourth- and fifth-year undergraduate dental students. Section A comprised four perception-based questions rated on a five-point Likert scale (1 = strongly agree, 5 = strongly disagree). Section B comprised two closed-ended questions assessing willingness to use the typodont teeth in future training. Tooth identities were anonymised during data collection. Tooth A = 15 wt.% carbonated hydroxyapatite composite; Tooth B = 15 wt.% Puraflake® composite; Tooth C = 15 wt.% zinc oxide composite; Tooth D = dental resin composite. Material identities were not disclosed to participants prior to or during questionnaire completion.

Material Characterisation

As these formulations were produced in-house rather than sourced commercially, no manufacturer datasheet values were available. Degree of conversion was measured by FTIR (Perkin-Elmer Frontier MIR/FIR spectrometer with an ATR unit, Perkin-Elmer Inc., USA), comparing the ratio of aliphatic C=C to aromatic C=C absorption peaks before and after curing. Vickers microhardness was measured

using a Buehler Micromet-4 microhardness tester (Buehler, USA), applying a 500 gf load with a dwell time of 10–15 s in accordance with ISO 6507/ASTM E384. Flexural modulus was measured by three-point bending in accordance with ISO 4049 (dental materials), using an Instron 5567 universal testing machine (Instron, UK). Filler particle size distribution was assessed using two particle size analysers to cover the full nanometre-to-micrometre range: a Zetasizer (0.1 nm–1 μm range) and a Mastersizer 2000 (0.3–1000 μm range) (both Malvern Instruments Ltd., UK). Eight replicate measurements (n = 8) were obtained per material for each test.

Table S1. Material characterisation summary for the four 3D-printed typodont formulations.

Material	DC (%)	Vickers Hardness (HV)	Flexural Modulus (GPa)	Filler Characteristic
Dental Resin Composite	58	21	1.9	4 wt.% zinc oxide filler, of the same order as the filler used in the Zinc Oxide group
Puraflake®	74	25	2.3	~5–40 μm lateral dimension; ~0.35–1 μm thickness (glass flake)
Zinc Oxide	70	22	2.0	~0.2–2 μm agglomerates
Carbonated Hydroxyapatite	72	21	1.9	~0.2–2 μm agglomerates

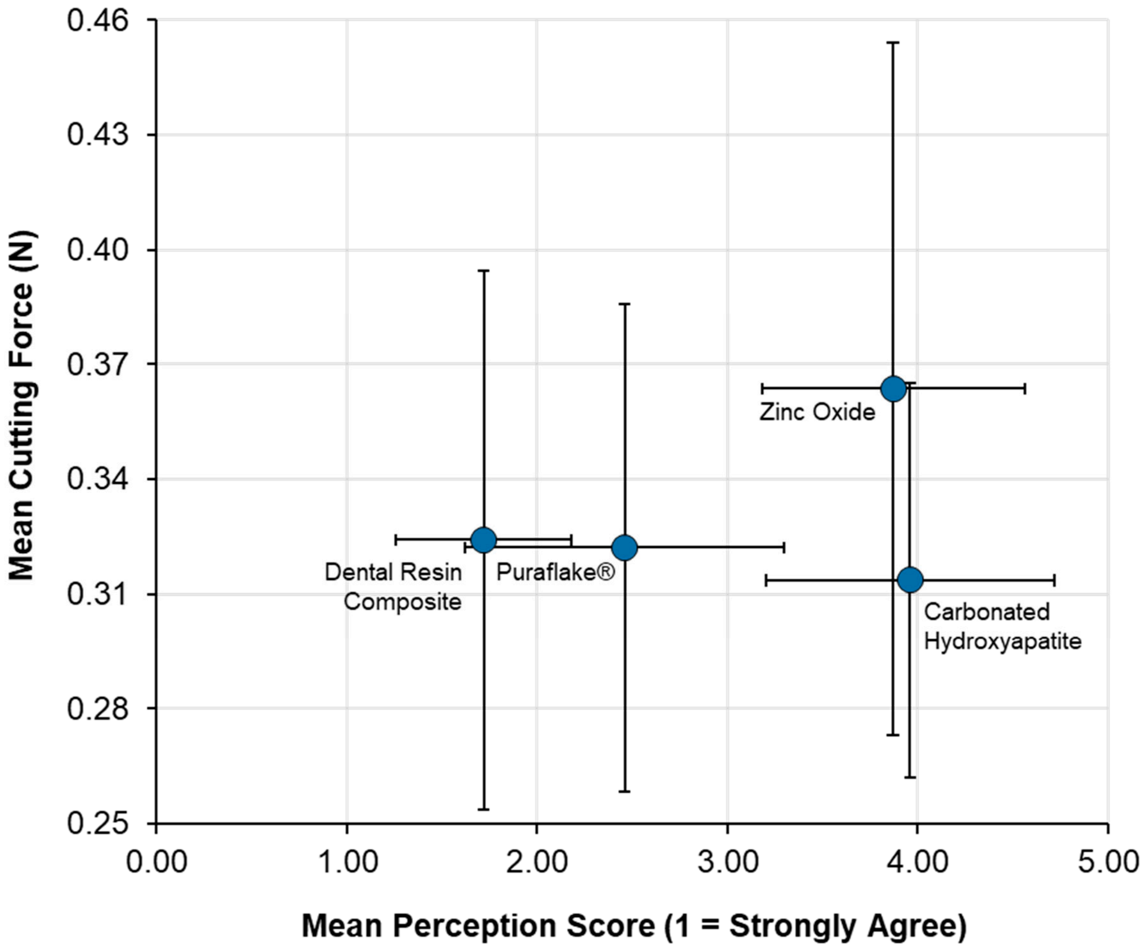

Figure S2. Relationship between mean cutting force (N) and mean Likert-scale perception score for the four 3D-printed typodont materials. Each point represents one material (n = 8 cutting force measurements; n = 46 perception ratings per material). Error bars represent ±1 standard deviation on both axes. No significant correlation was observed between cutting force and perception score (Spearman $\rho = -0.40$, $p = 0.60$), indicating that objective force equivalence does not predict perceived haptic similarity to extracted enamel. Lower perception scores indicate greater perceived similarity to enamel.

Free-Text Comments Themes

Theme 1: Perceived realism of cutting behaviour

Several respondents commented on the cutting response of the 3D-printed typodont teeth, particularly in relation to how closely the materials resembled extracted enamel during drilling. The dental composite resin was most frequently noted as providing a more realistic cutting experience.

- "The composite tooth felt closer to drilling a real tooth compared to the others."
- "Resistance felt more natural when cutting, similar to extracted teeth."
- "The composite material behaved more like enamel when starting the cavity."

In contrast, some respondents noted differences in surface behaviour for other materials.

- “One of the teeth felt slightly more brittle when drilling.”
- “Some materials produced a different surface feel compared to real enamel.”

Theme 2: Comparison with commercial typodont teeth

Students commonly compared the 3D-printed typodont teeth with the commercial typodonts used routinely in pre-clinical teaching, often favouring the printed models.

- “These felt more realistic than the plastic teeth we normally use.”
- “The standard typodonts feel harder and less like real teeth.”
- “The 3D-printed teeth were easier to control during drilling than the commercial ones.”

Theme 3: Educational value and future use

Respondents highlighted the perceived usefulness of the 3D-printed typodont teeth for undergraduate training and expressed willingness to use them again in future teaching sessions.

- “I would be happy to use these again in pre-clinical sessions.”
- “These would be useful earlier in the course before treating patients.”
- “I would prefer to practise on these rather than the current typodont teeth.”
